# Combinatorial synthesis of heteroepitaxial, multi-cation, thin-films via pulsed laser deposition coupled with in-situ, chemical and structural characterization

**DOI:** 10.1038/s41598-022-06955-5

**Published:** 2022-03-07

**Authors:** E. J. Moon, A. Goyal

**Affiliations:** 1 Laboratory for Heteroepitaxial Growth of Functional Materials & Devices, Department of Chemical & Biological Engineering, State University of New York, Buffalo, NY 14260 USA; 2 Now at, Corning Incorporated, Corning, NY 14830 USA

**Keywords:** Materials science, Materials for devices, Materials for energy and catalysis, Techniques and instrumentation

## Abstract

Combinatorial synthesis via a continuous composition spread is an excellent route to develop thin-film libraries as it is both time- and cost-efficient. Creating libraries of functional, multicomponent, complex oxide films requires excellent control over the synthesis parameters combined with high-throughput analytical feedback. A reliable, high-throughput, in-situ characterization analysis method is required to meet the crucial need to rapidly screen materials libraries. Here, we report on the combination of two in-situ techniques—(a) Reflection high-energy electron diffraction (RHEED) for heteroepitaxial characterization and a newly developed compositional analysis technique, low-angle x-ray spectroscopy (LAXS), to map the chemical composition profile of combinatorial heteroepitaxial complex oxide films deposited using a continuous composition spread method via pulsed laser deposition. This is accomplished using a unique state-of-the-art combinatorial growth system with a fully synchronized four-axis mechanical substrate stage without shadow masks, alternating acquisition of chemical compositional data using LAXS at various different positions on the $\sim$ 41 mm $\times$ 41 mm range and sequential deposition of multilayers of SrTiO$_3$ and $\hbox {SrTi}_{0.8}\hbox {Ru}_{0.2}\hbox {O}_3$ on a 2-inch (50.8 mm) $\hbox {LaAlO}_3$ wafer in a single growth run. Rutherford backscattering spectrometry (RBS) is used to calibrate and validate the compositions determined by LAXS. This study shows the feasibility of combinatorial synthesis of heteroepitaxial, functional complex oxide films at wafer-scale via two essential in-situ characterization tools—RHEED for structural analysis or heteroepitaxy and LAXS for compositional characterization. This is a powerful technique for development of new films with optimized heteroepitaxy and composition.

## Introduction

Combinatorial synthesis is crucial for the discovery of novel materials for next-generation applications and for developing material libraries^1–9^. Combinatorial approaches are designed to enhance efficiency in materials science research by efficiently fabricating, processing, and surveying of a vast number of materials combinations suggested via theory and computational modeling^10–15^. However, high-throughput experimental techniques, such as fully automated, wafer-scale synthesis of advanced complex materials film with screenable characterization techniques to characterize the high volume of samples generated, still need further development. Pulsed laser deposition (PLD) has been previously used in combinatorial growth via continuous composition spread (CCS) synthesis^16–18^. Heteroepitaxial films of complex functional oxides can be grown using PLD, a process that delivers a fast, atomic-scale growth from different multi-cation and multi-component targets in a single run^16,19^. PLD-based CCS technique can produce material libraries of functional complex oxide films using lateral thickness gradients across wafer-scale substrates by sub-monolayer sequential deposition of the different constituents under precisely controlled growth parameters followed by interdiffusion between the sub-monolayers.

Strongly correlated electron systems have been shown to exhibit a wide range of interesting physical phenomena, such as high-T$_\texttt {c}$ superconductivity, multiferroicity, high mobility electron gases, and giant magnetoresistance, that demand both fundamental research and applied research to address various applications^20–22^. Complex oxides sensitive to the various degrees of freedom can be tuned by dopant, cation ordering, layer thickness, strain, and interfacial engineering^23,24^. However, synthesis of high-quality and high-performance complex oxide films on a large scale is challenging. It requires sophisticated control of thermodynamical stability-, single-phase crystallinity-, and stoichiometric complexity- governed by the element-specific kinetics and dynamics under various growth conditions. It requires extensive calibration process loops to identify optimal growth parameters. In addition, this needs to be combined with compositional tuning of multicomponent complex oxide films to study a range of physics phenomena that varies with composition. The combination of process variables, compositional tuning, heteroepitaxy or structural tuning results in a very large process space that requires significant time and resource-intensive experimentation.

The discovery of new materials and the development of innovative technological devices and applications will be significantly enhanced with material libraries fabricated using a PLD-based combinatorial synthesis process (as it allows for a high degree of control over compositions of multicomponent film) combined with rapid in-situ analytical tools.

A rapid analytical technique that probes chemical concentration with a high-spatial resolution over a CCS film deposited on a large diameter wafer or substrate is critical for identifying the optimal material composition for a specific application. Efforts to develop physical characterization methods have been reported, for instance, temperature- and thickness-dependent structure evolution utilizing x-ray diffraction (XRD) images of $\hbox {BaTiO}_3/\hbox {SrTiO}_3$ multilayer films on a $\hbox {SrTiO}_3$ by controlling mask configuration, local electronic states probed by in-situ low-temperature scanning tunneling microscopy (LT-STM) of FeSe film on a LiF utilizing a controlled mask for gradient thickness, and atomic-scale phase evolution probed by atomic probe tomography (APT) of Cr-Mn-Ni-Co-Fe sources coated a Si tip array, and so on^25–28^. These techniques are either ex-situ or in-situ post-growth processes with transferring samples to a separate analysis chamber that causes long turn-around times. We have found no published reports of in-situ, high-throughput analytical techniques that can rapidly scan wafer-scale samples to characterize their chemical composition during the growth of complex, multicomponent heteroepitaxial films.

**Figure 1:**
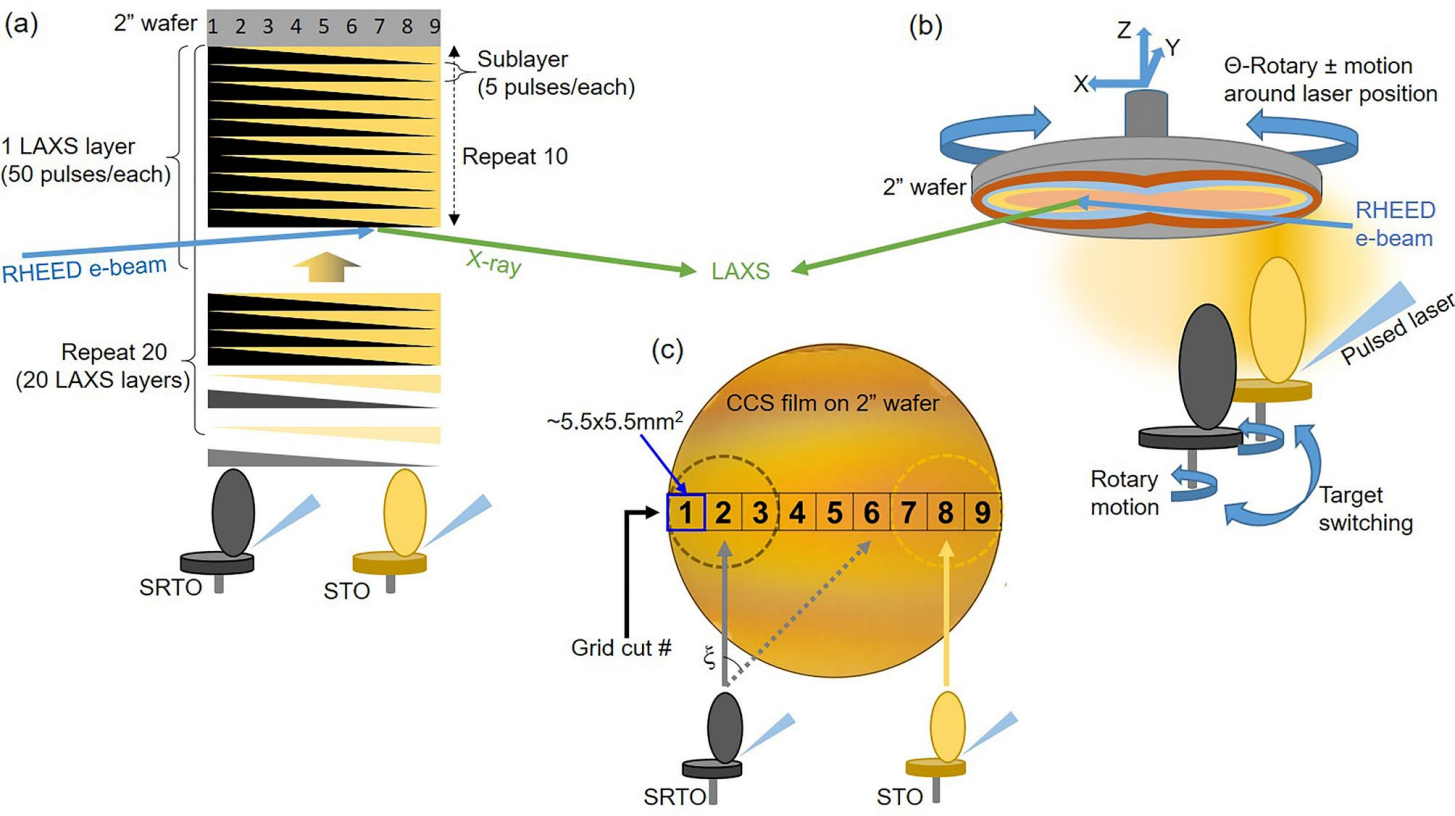
Illustration of the combinatorial synthesis configuration used for the hetero-epitaxial growth utilizing a PLD equipped with in-situ high throughput analytical technique. (**a**) CCS film growth strategy with alternatively performing the multilayers deposition of sub-unit-cell thick layers of each target (e.g., $\hbox {SrTiO}_3$ and $\hbox {Sr(Ti,Ru)O}_3$) and LAXS measurement. (**b**) In-situ PLD-based CCS-LAXS mechanical configuration with a four-axis mechanical substrate stage (X, Y, Z, and $\Theta$ (rotation)) and a three-rotation setup for targets switch and raster to fixed laser strike location. (**c**) CCS film deposited on a 2-inch diameter wafer showing colored contour. The selected 9 grids cut by $\sim$ $5.5\times 5.5\hbox { mm}^2$ labeled from #1 to #9, from STRO deposition side to STO, respectively.

A newly developed technique, low-angle x-ray spectrometry (LAXS) measures the characteristic x-rays emitted from a solid sample by the electron beam of a reflection high-energy electron diffraction (RHEED) already present in MBE growth systems^29,30^. This previous work demonstrated the technique’s reliability as an in-situ composition measurement tool on amorphous films utilizing three metal targets^29,30^. However, no previous work has demonstrated using both structural (RHEED) and chemical analysis (LAXS) which is needed for growth of heteroepitaxial, combinatorial films. In such films, validation of heteroepitaxial growth as a function of composition is determined using RHEED and the exact compositional analysis is done using the LAXS technique.

In this study, we demonstrate a PLD-based high-throughput combinatorial synthesis of hetero-epitaxial functional films via sequential, layer-by-layer deposition utilizing an in-situ, high-throughput, wafer-scale, structural and compositional analysis. We report studies on two model systems with elevated doping levels to demonstrate a pseudobinary, heteroepitaxial CCS film of a well-known class of materials, $\hbox {SrTiO}_3$ (STO) and $\hbox {SrTi}_{0.8}\hbox {Ru}_{0.2}\hbox {O}_{3-\delta }$ (STRO), deposited on $\hbox {LaAlO}_3$ (LAO) wafers.

## Results and discussion

Figure 1 illustrates the combinatorial synthesis configuration of the PLD-based CCS-LAXS growth system used in this study. The CCS film growth strategy includes multilayers comprising one full growth with brief growth interruptions 20 times for compositional scans using the LAXS technique on a wafer-scale substrate. The detailed CCS process routines adopted in this study are described in the “Methods” section. The CCS sample of 2-in diameter cut into by $\sim$ 5.5 $\times$ 5.5 $\hbox { mm}^2$ grids labeled from #1 to #9, starting from the STRO deposition side to the STO side, for subsequent detailed XRD and RBS measurements. X-ray diffraction was conducted to determine film quality and thickness. The calibration and validation of the cation composition profile (Ti/Sr and Ru/Sr), along with $\hbox {SrTi}_{1-x}\hbox {Ru}_x\hbox {O}_{3-\delta }$, of the analysis by the LAXS algorithm was supported by RBS.

**Figure 2:**
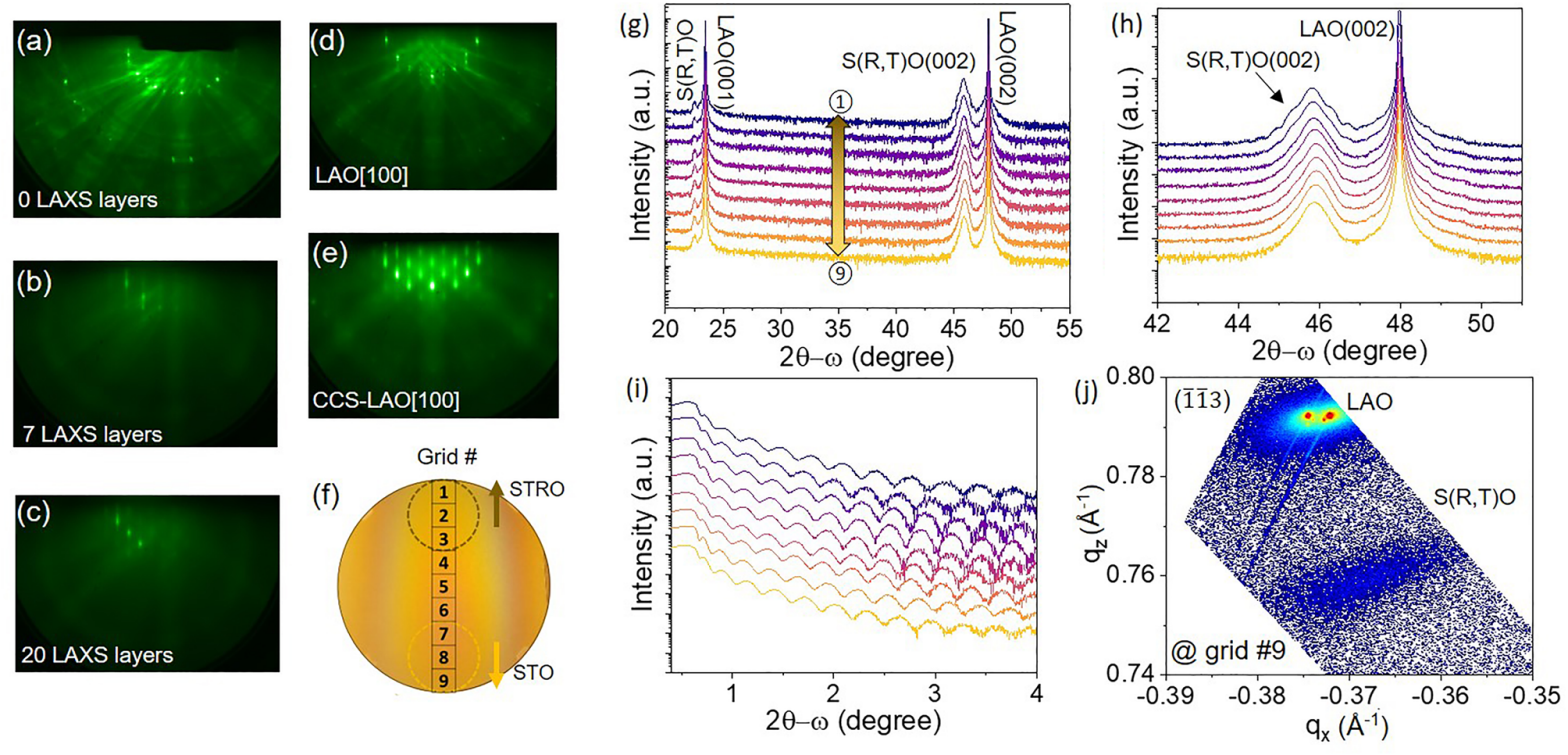
(**a**) Evolution of the RHEED pattern at different stages of the CCS growth, for the film thickness of 0 LAXS layer (0 laser pulses), (**b**) 7 LAXS layers (total 700 laser pulses), and (**c**) 20 LAXS layers (total 2000 laser pulses). (**d**) RHEED pattern of the 2D terminated LAO wafer before deposition (along LAO(001) [100] azimuthal direction) and (**e**) the corresponding image after CCS film deposition. (**f**) The CCS film on a 2-inch diameter LAO wafer displaying the selected 9 grids labeling from #1 to #9 across the two target deposition locations. (**g**) XRD 2- scans on the nine grid squares. (**h**) $2\theta -\omega$ (002) Bragg peak, (**i**) Corresponding x-ray reflectivity scans. (**j**) Reciprocal space map ($\bar{1}\bar{1}3$) reflection at the grid #9.

In-situ high-pressure RHEED was used not only to generate the data acquired by the CCS-LAXS system but also to monitor the surface structure of the growth. RHEED images of the CCS film on the LAO substrate at different deposition stages are shown in Fig. 2a–c. Figure 2a shows the bare LAO wafer with numerous Kikuchi lines, indicating the high crystallographic quality of the surface. It is noted that the images were taken where the wafer was at the rotation angle for STO deposition (rotary angle of a substrate stage, $\Theta =225^{\circ }$). The CCS film initially grew in the 2D mode with a streaky pattern, as indicated by the RHEED image shown in Fig. 2b. After 16 measurement cycles using the LAXS, a 3D mode appeared (total 1600/2000 laser pulses), and it kept this form until the end (total 2000 laser pulses) of the process (Fig. 2c). Figure 2d shows the RHEED patterns from an LAO wafer in high symmetry azimuthal directions before the growth process began, and the pattern of the CCS film after the growth was completed is shown in Fig. 2e. The final deposited CCS film according to the evolution of the RHEED images indicates a slightly rough surface, however the film is still heteroepitaxial.

To elucidate the detailed epitaxial quality of the CCS film, a standard 4-circle XRD was performed ex-situ. Figure 2f shows the location of nine grid squares on the 2-inch diameter LAO wafer within which XRD measurements were made. Figure 2g shows the $2\theta -\omega$ wide scan made on each grid square displayed from #1 (near the STRO deposition center side) through to #9 (near the STO deposition center side). The results show only the (00*l*) Bragg peaks, indicating the c-axis orientation of the film along [00*l*] wafer crystallographic direction, with no secondary and impurity phase peaks of (110) and (111) within the scan range. It confirms that the CCS film on the LAO wafer is heteroepitaxially grown. The corresponding $\theta -\omega$ around (002) reflection of the CCS films are shown in Fig. 2h. The (002) film peak does not exhibit clear fringes which are expected from the evolution of the RHEED pattern. Figure 2i shows experimental reflectivity results. The thickness variation of the CCS films is expected to change across the wafer by a factor of $\hbox {cos}^n\xi$, where $\xi$ is the angle in the laser plume propagating direction from a target, as shown in Fig. 1c^19^. Figure 2j shows reciprocal space map (RSM) scanned at grid square #9 as a function of the out-of-plane momentum transfer vector, $\hbox {q}_z$ and in-plane, $\hbox {q}_x$, which reveals an elongated film peak. Taken together, the XRD results suggest that the film could be in-homogeneously strained (see Fig. S1).

**Figure 3:**
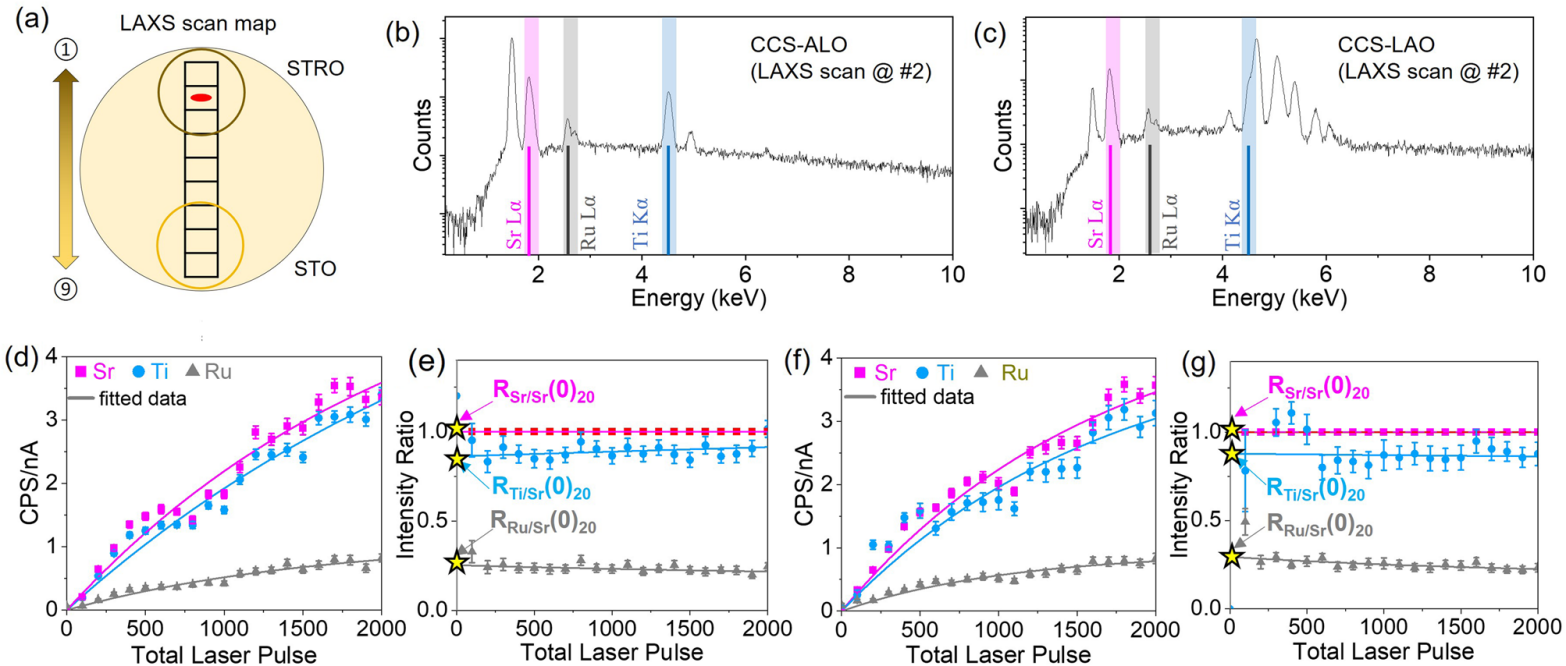
(**a**) A diagram showing LAXS scan locations from #1 to #9, from STRO to STO side, respectively. X-ray line intensity spectrum scanned at the selected LAXS scan location #2 (shown in red) of a CCS film on a sapphire (hereafter, ALO) wafer (**b**) and an LAO (**c**). The x-ray lines of interest elements (Sr $\hbox {L}_{\alpha }$, Ti $\hbox {K}_{\alpha }$, and Ru $\hbox {L}_{\alpha }$) are highlighted. X-ray line intensities of three elements as a function of laser pulse number from CCS film on ALO (**d**) and LAO (*f*). X-ray line intensity ratios ($\hbox {R}_{\text {EOI/Sr}}$(*t*)) to the Sr $\hbox {L}_{\alpha }$ line intensity as a function of laser pulse number of CCS film on ALO (**e**) and LAO (**g**). Note that “EOI” is element of interest (Ti and Ru) and “*t*” is deposited film thickness or total laser pulses at LAXS acquisition. $\hbox {R}_{\text {EOI/Sr}}$(0) marked by yellow stars on a x-axis in (**e**) and (**g**) indicates the value at *t*=0 of the fitted relative intensity.

**Figure 4:**
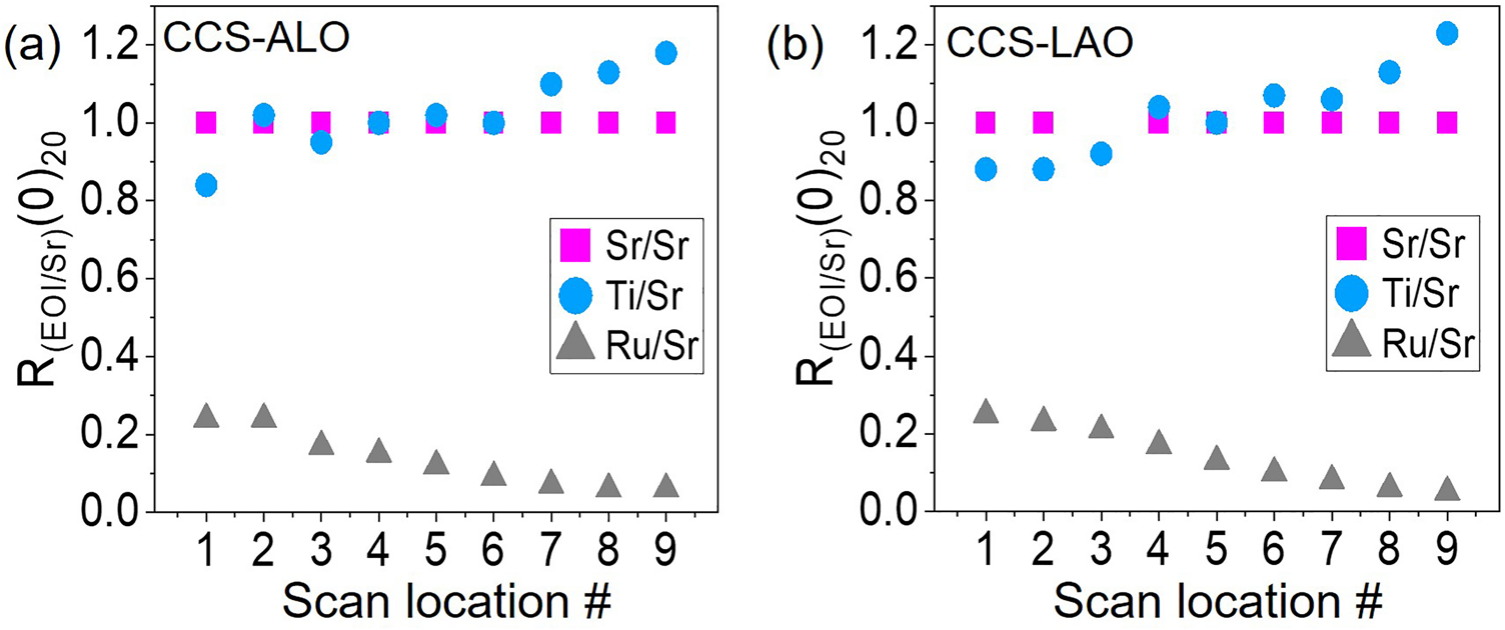
Intensity ratio profile ($\hbox {R}_{\text {EOI/Sr}}(0)_{20}$) of CCS film as relative elemental content to Sr as a function of the scanning position on the CCS/ALO (**a**) and the CCS/LAO (**b**). $\hbox {R}_{\text {EOI/Sr}}(0)_{20}$ indicates $\hbox {R}_{\text {EOI/Sr}}(0)$ obtained when the 20-CCS-LAXS process completed.

The data acquisition procedure for the CCS growth using LAXS analysis is shown in Fig. 3. The LAXS measurements were performed on the selected 9 scan locations in each of 20 CCS-LAXS cycles during multilayer deposition process that occurred in a single growth. Figure 3a shows a schematic image of LAXS scan locations index from #1 to #9, in the direction from STRO to STO. Figure 3b,c show the x-ray characteristic spectrum of the CCS films on sapphire (hereafter, ALO) and LAO wafers at the scan location #2 (red oval in Fig. 3a). The x-ray lines of the elements (e.g., Sr $\hbox {L}_{\alpha }$, Ti $\hbox {K}_{\alpha }$, and Ru $\hbox {L}_{\alpha }$) are highlighted and marked, at 1.806 keV, 4.508 keV, and 2.558 keV, respectively. Figure 3d,f show the corresponding x-ray line intensities obtained from Fig. 3b,c as a function of the laser pulses, which are normalized to the electron beam current. The x-ray line intensity ratios of element pairs (Sr/Sr, Ti/Sr, and Ru/Sr) as a function of laser pulse number are shown in Fig. 3e,g. The data set of 3 elements at each cycle was obtained by LAXS measurement, and the solid lines are the line of best fit to the measured data. This study focuses on the composition gradient of *B*-site cations (Ti and Ru) in the combinatorial layer-by-layer growth of STO and Sr(Ti,Ru)O targets. Note that the x-ray line intensity ratios are defined by $\hbox {R}_{\text {EOI/Sr}}(t)_n$, where *t* represents film thickness (which is a function of the number of laser pulses) and *n* indicates the total CCS cycles (LAXS layers). The ratio $\hbox {R}_{\text {EOI/Sr}}(0)_{20}$ indicates the R-value at *t* = 0 (intercept point on the y-axis at an x-axis value of zero) achieved from the fitted dataset of all 20 CCS-LAXS cycles. Through this process, the absorption and fluorescence re-correction factors become negligible as *t* $\rightarrow$ 0.

**Figure 5:**
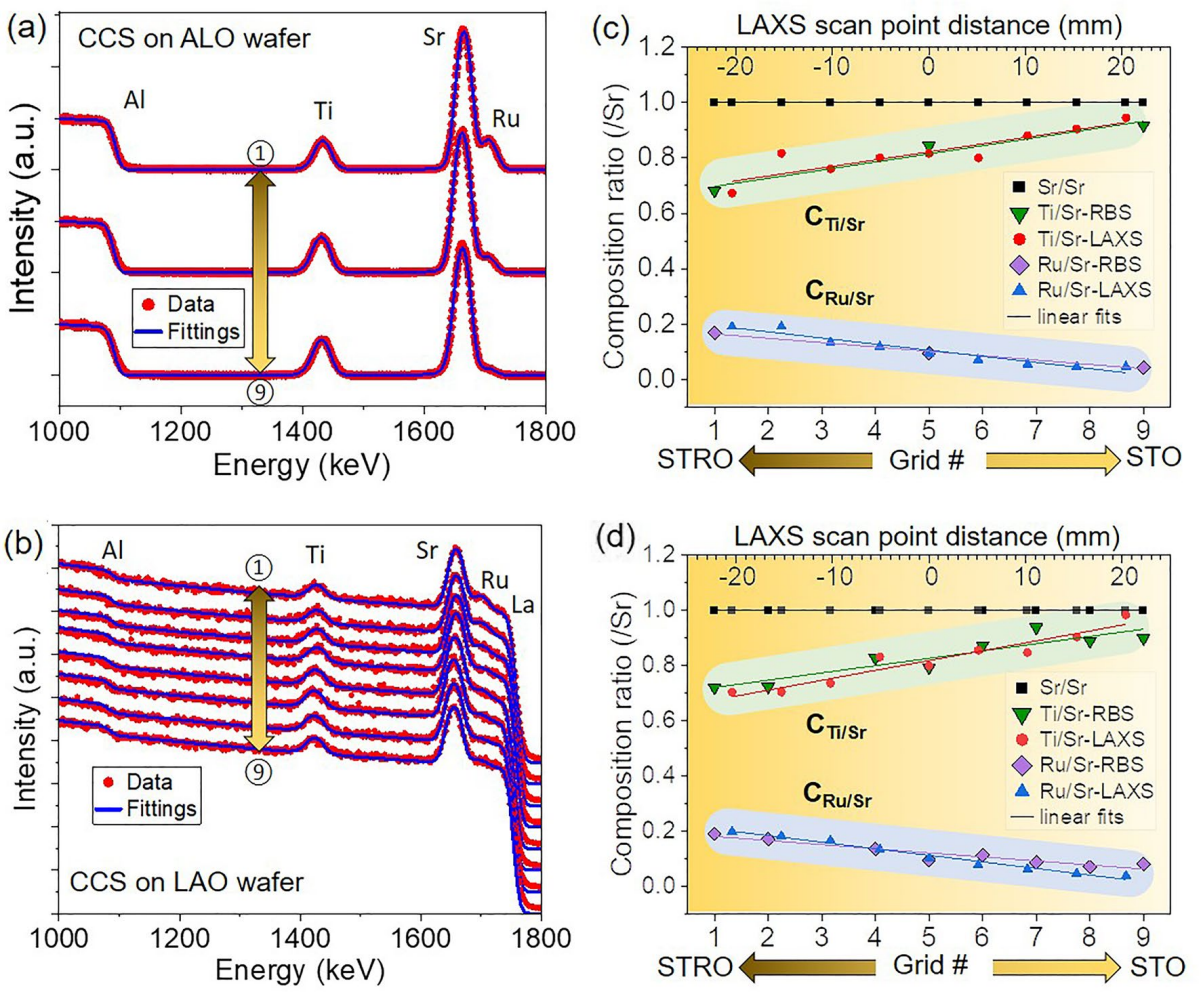
Experimental RBS spectra (**a**) at three different locations (grids #1, #5, and #9) of a CCS film grown on a ALO wafer and (**b**) at eight different locations (grids #1–#9, except #3) of a CCS film grown on an LAO wafer, along with the optimized SIMNRA simulations following by fittings to obtain the film composition. (**c**) Relative composition ratio to Sr, obtained from (**a**), and the CCS-LAXS relative composition profile adjusted by sensitivity factors achieved as a function of position on the wafer. (**d**) Relative composition ratio obtained from (**b**), and the CCS-LAXS relative composition profile adjusted by sensitivity factors as a function of the position of the wafer. Note that the upper x-axis in (**c**) and (**d**) indicate the distance of LAXS scan locations from #1 to #9 across the wafer in millimeters (mm). Grid #5 and LAXS scan location #5 are both located at the wafer’s center point.

Figure 4a,b show corresponding x-ray line intensity ratios ($\hbox {R}_{\text {EOI/Sr}}(0)_{20}$) obtained from the process shown in Fig. 3 as a function of the scan location over the CCS films on ALO and LAO wafers. Note that the LAXS scanning points # number associated locations can be found in Fig. 3a. The Ru relative ratio profile to Sr is at a maximum at #1, near the STRO deposition center and gradually decreases in the direction of #9. The maximum relative ratio of Ti to Sr is located at #9 and decreases as the scan position moves to #1. These relative intensity profile gradients of Ti and Ru are consistent with the PLD deposition geometry and deposition rates (see Fig. 1). The R ($t \rightarrow$ 0) value can be directly proportional to the film’s chemical composition in accordance with the ZAF effect correction as reported recently^29,30^. Therefore, the relative intensity can be defined by $\hbox {R}_{\text {EOI/Sr}}$ ($t \rightarrow$ 0) = ($\hbox {C}_{\text {EOI}}/\hbox {C}_{\text {Sr}}$)$\times$($\hbox {K}_{\text {Sr}}/\hbox {K}_{\text {EOI}}$), where the relative sensitivity factor $\hbox {K}_{\text {Sr}}/\hbox {K}_{\text {EOI}}$ is a constant if the elements combination and experimental setup are the same. Indeed, the composition ratio ($\hbox {C}_{\text {EOI}}/\hbox {C}_{\text {Sr}}$) can be obtained by $\hbox {R}_{\text {EOI/Sr}}$ ($t \rightarrow 0)_{20} \times (\hbox {K}_{\text {EOI}}/\hbox {K}_{\text {Sr}}$). The relative sensitivity factor ($\hbox {K}_{\text {EOI}}/\hbox {K}_{\text {Sr}}$) can be determined by one-time calibration via Rutherford backscattering analyses.

To investigate the composition of sample arrays and to verify the sensitivity factor for the composition ratio calibration of the LAXS analysis, we performed RBS at the nine grids across the two target deposition points on the CCS films grown on the 2-inch LAO and ALO wafers. SIMNRA simulation software was used to model experimental RBS data for the composition profiles of a series of samples^31^. Figure 5a shows the RBS spectra of the CCS films on an ALO wafer and the optimized simulated and fitted spectra. The Ru peaks in grid #1 (near the STRO target side), #5 (center position of the wafers in between the two targets), and #9 (near the STO target side) are distinguishably reduced. The RBS spectra of the CCS film on the LAO wafer are shown along with the fitted spectra in Fig. 5b. Note that the sample at grid #3 is not included in this arrays, whereas the rest eight sample arrays were simulated and fitted. The relative concentration ratios of Ti and Ru to Sr obtained by the RBS analysis of the CCS film on ALO and the epitaxially grown CCS film on LAO are shown in Fig. 5c,d. Note that the gap between adjacent LAXS scan locations is $\sim 5.14$ mm, whereas the gap between Grids is $\sim \hbox {5.5 mm}$. Grid #5 and LAXS scan location #5 are both located at the wafer’s center point. The trend in the composition ratios obtained from RBS analysis, $\hbox {C}_{\text {Ti/Sr-RBS}}$ and $\hbox {C}_{\text {Ru/Sr-RBS}}$, is consistent with the PLD-based CCS growth configuration. The compositional profile from the LAXS analytical data, when calibrated by a sensitivity factor, and the RBS data are shown in Fig. 5c,d for the polycrystalline CCS sample and the hetero-epitaxial CCS film, respectively. The sensitivity factor ($\hbox {K}_{\text {Ti/Sr}}$ and $\hbox {K}_{\text {Ru/Sr}}$) obtained from the RBS data was 0.8. There is a good agreement between the RBS and the calibrated LAXS data. The trend in the composition data indicates that in-situ LAXS analysis can be utilized as a high-throughput processing and screening tool for large-scale CCS functional film synthesized by PLD.

## Conclusion

This study shows that combinatorial synthesis of heteroepitaxial, complex, multicomponent oxide films at a wafer-scale combined with in-situ, rapid feedback analysis is a powerful materials optimization technique. Using the CCS-PLD technique heteroepitaxial films can be deposited on wafer-scale substrates with varying chemical compositions, deposition rates and layer thicknesses. The programmable CCS loop controls both the automated sequential combinatorial synthesis and the scanning x-ray spectrometry undertaken at numerous locations in a single run. Integrated multiple linear shifts and a rotary mechanism for X–Y–Z–$\Theta$ positioning of the sample manipulator are used to place the CCS-LAXS sample in the desired location for combinatorial growth and the chemical composition interrogation. The X–Y linear shift modular can range up to $\sim$ 41 mm in the X and Y directions with $\sim 10\,\upmu \hbox {m}$ precision. The corresponding RBS results verify that the LAXS analytic algorithm package works well as an in-situ compositional feedback technique. In-situ RHEED (and ex-situ XRD) on the CCS-LAXS films establish the quality of the heteroepitaxial films at each location or composition. This study reports in-situ, simultaneous, compositional, high-throughput characterization of heteroepitaxial films in a single growth run, for the first time. Such an approach allows rapid identification of optimal compositions for heteroepitaxial film growth. When combined with depositions at different growth temperatures, it allows rapid mapping of conditions for heteroepitaxial film growth, including film composition. Such combinatorial growth with high-throughput in-situ compositional feedback and in-situ structural feedback can significantly benefit materials design and innovation, and accelerate development of next-generation materials and devices.

## Methods

### PLD-based composition continuous spread synthesis

The two targets, ${\text{SrTiO}}_3$ and Ru-doped ${\text{SrTiO}}_3$ (${\text{Sr}}{({\text{Ti}},{\text{Ru}})}{\text{O}}_3$), were used to create a lateral binary phase spread. These targets were pointed on the opposite side of 2-inch diameter wafers, e.g., ${\text{LaAlO}}_3$ and *r*-plane sapphire wafers, and deposited by a pulsed laser deposition system (Neocera Pioneer 180 PLD). The system is equipped with in-situ RHEED and LAXS (PLD-based CCS-LAXS system). The wafer was positioned relative to the target in such a way that the plume from the target deposited material on the wafer’s edge (Fig. 1a). Also, the lateral inhomogeneity of the typical PLD deposition gives thickness variation of CCS films as a function of the distance from the plasma plume, without various mask configurations. Figure 1b shows the CCS-LAXS combinatorial growth configuration that operates a sequential four-axis mechanical substrate stage (X, Y, Z, and $\Theta$ (rotation)) and a three-rotation setup for targets switch and raster fixed laser strike location. The substrate rotates by $180^{\circ }$ after each target deposition so that the different target deposition can be distinguished, simultaneously with the synchronized movement of targets’ switching after each sub-monolayer deposition. The CCS synthesis was made in an oxygen partial pressure of 100 mTorr at a laser fluence $\sim 1.7\hbox { J/cm}^2$ at the target surface of 5 Hz repetition for both targets by pulsed laser beams of 248 nm wavelength (Coherent COMPex Pro 120 KrF excimer laser). The distance between a target and a sample is $\sim$ 60 mm. During deposition, the wafer temperature was held at $\sim$ 650 $^{\circ }\hbox {C}$. The oxygen back-fill pressure while cooling after the CCS-LAXS synthesis process was 400 Torr.

### Film structural analysis

Film growth was monitored using in-situ high-pressure RHEED. X-ray diffraction (XRD) was carried out using a Panalytical Empyrean diffractometer equipped with a $2\times \hbox {Ge(220)}$ incident beam hybrid monochromator using Cu $\hbox {K}\alpha _1$ radiation. A hybrid detector ($\hbox {PIXcel}^{\text {3D}}$) and a point detector (proportional detector) with an attenuator were used for sample alignment and measurements depending on each acquisition condition. The samples were cut by $\sim$ 5.5$\times$5.5 mm$^2$ from a 2-inch diameter of sapphire and LaAlO$_3$ wafers for the ex-situ post-growth characterization. Reciprocal space maps (RSM) around ($\bar{1}\bar{1}3$) were scanned on the samples of the selected 9 grids located between the two target deposition areas of an LAO wafer.

### High throughput composition profile analysis

A low-angle x-ray spectrometer (LAXS) performed in-situ composition analysis during film growth. The electron beam (30 keV) of a high-pressure RHEED is used to generate x-rays from a sample and the high sensitivity silicon drift detector of a LAXS measures the x-rays^29,30^. The output data for the compositional profile is adjusted by a unique algorithm in the LAXS package to eliminate the ZAF effect^29,30^. The incident angle of the electron beam from RHEED is $\sim 4.5^{\circ }$ relative to the film surface, and the x-ray detector of the LAXS is pointed at $\sim 10^{\circ }$ relative to the film surface. LAXS is capable of scanning locations at 37 regions (Fig. S3) within a 2-inch wafer at locations selected by the system software package. However, in this study, nine locations were scanned by LAXS. Each programmed routine of LAXS data-acquisition and CCS-growth in a single CCS-LAXS loop includes the following steps: (1) a layer-by-layer deposition of sub-unit-cell from each target by laser ablation, (2) a substrate stage movement in X-Y axis for $\sim \hbox {3 mm/s}$ to the initial scan position of LAXS after pausing the deposition, (3) LAXS measurement on the selected scan locations, (4) resumed deposition back to the location at which step (1) began, and (5) LAXS on the scan locations. For each LAXS layer, one cycle consists of steps (1) to (3). These 3 steps repeat as programmed, in a single growth loop. We ran a total of 20 cycles (20 LAXS layers). Each LAXS scan was set to stop when the count within the ROI region reaches at 3000 counts or 40 seconds. Each LAXS cycle (9 scans) took approximately $\sim$ 3–6 total minutes for scanning all the 9 points. The final output data of the LAXS package is reported as a composition profile with a sensitivity factor that requires one-time calibration. RBS was used for calibration and validation of the LAXS processed data. RBS measurements were performed at the RBS facility of the Laboratory for Surface Modification at Rutgers University utilizing the General Ionex tandem accelerator, running $\hbox {He}^{++}$ ions at 2 MeV. The measurement geometry was chosen to consist of an incident beam of $0^{\circ }$, an exit angle of $17^{\circ }$, and a scattering angle of $163^{\circ }$, respectively, to achieve better depth and mass resolution, and minimal broadening. SIMNRA simulation software was used to model the experimental RBS data for the composition profiles^31^.

## Supplementary Information

Supplementary Information.
