# Supplementary material for: Combinatorial synthesis of heteroepitaxial, multi-cation, thin-films via pulsed laser deposition coupled with in-situ, chemical and structural characterization
Source: Sci Rep. 2022 Mar 7;12:3219. doi: 10.1038/s41598-022-06955-5 (PMC8901668; doi:10.1038/s41598-022-06955-5)
Supplement: Supplementary file 1 — Supplementary Information. [file 41598_2022_6955_MOESM1_ESM.pdf]

Supplemental Information

E. J. Moon[1,2] and A. Goyal[1,+]

[1]Laboratory of Heteroepitaxial Growth of Functional Materials Devices, Department of Chemical and Biological Engineering, State University of New York, Buffalo, NY, 14260 USA
[2]Research and Education in eNergy, Environment and Water (RENEW) Institute, State University of New York, Buffalo, NY, 14260 USA
[+]agoyal@buffalo.edu

X-ray diffraction was used to measure c-axis parameters of $SrTiO_3$ (STO) films deposited on STO and LSAT ($La_{0.18}Sr_{0.82}Al_{0.59}Ta_{0.41}O_3$). The Grid #9 piece out of nine CCS film Grids of $Sr(Ti,Ru)O_3$ on LAO ($LaAlO_3$) is compared with the other two films because of the lowest Ru doping level (~4%) according to the LAXS analysis. Figure S1 (a) shows XRD measurements taken around the (0 0 2) truncation rod of the films grown on the three substrates. The film peaks exhibit noticeable shifts from the bulk STO lattice constant (a = 3.905 Å). The out-of lattice parameters of the films are displayed in Figure S1 (b), obtained from Figure S1(a). Based on the positions of the film (002) and the substrate of STO, LSAT, and LAO (002) reflections, the lattice parameters of the thin films are shifted in response to the various strain states. To elucidate the strain state of the CCS film, a reciprocal space map (RSM) around the $(\bar{1}\bar{1}3)$ Bragg peak for the CCS #9 film on LAO is shown in Figure S1 (c), taken from Figure 2 (j) of the text. The red vertical line indicates the in-plane (q_x) position of the CCS film and LAO exhibiting the same in-plane lattice constant. The yellow oval displays the elongated film peak spreading from the in-plane position. These XRD results reveal that the film could be inhomogeneously strained.

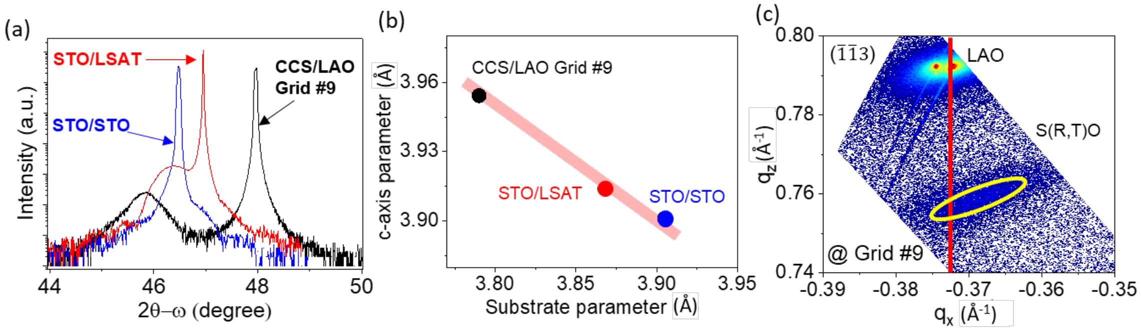

Figure S1. (Color Online) (*a*) XRD data for STO films on the different substrates and CCS Grid #9 on LAO. (*b*) The out-of-plane lattice parameters of the films as a function of the substrate lattice parameter. (*c*) Reciprocal space map measured from a CCS film (Grid #9) grown on LAO around the $(\bar{1}\bar{1}3)$ reflection.

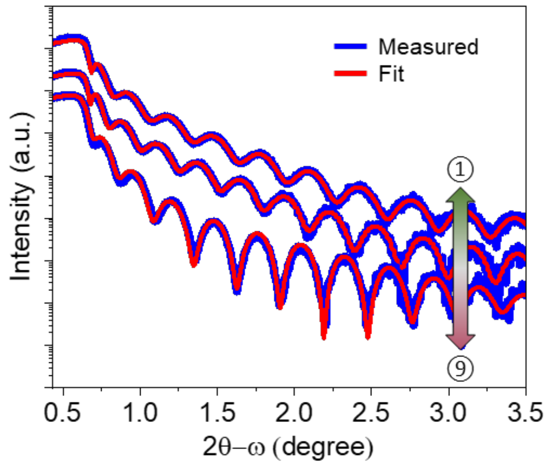

Figure S2. (Color Online) X-ray reflectivity (measured and fitting data) of Grid #1, #5, and #9 (from top).

X-ray reflectivity measurements were performed to determine the film thickness of the film. Figure S2 shows experimental reflectivity curves and fits of the 3 Grids labeled from STRO to STO side. The fits of the reflectivity curve were performed with the GenX program package (Ref. 30). The thicknesses are 278, 306, and 294 Å (#1, #5, and #9, respectively).

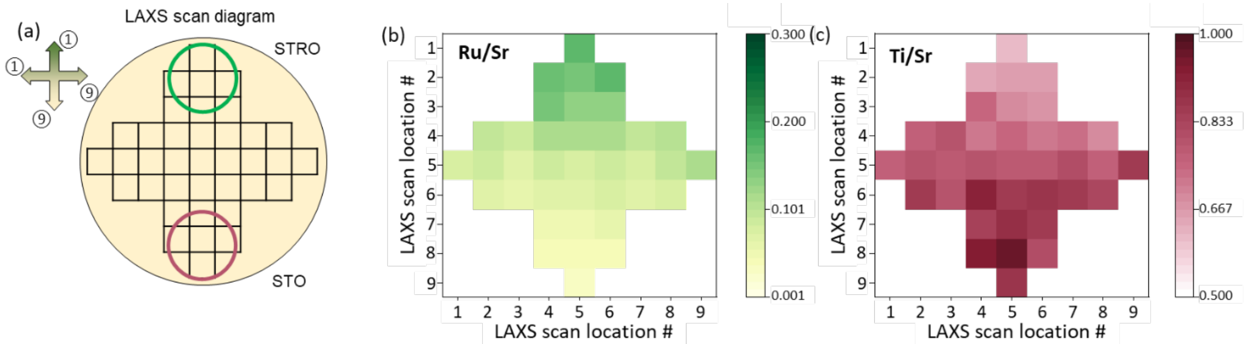

Figure S3. (Color Online) (*a*) 37 LAXS scan positions. The composition ratio of the full LAXS scan locations over a 2-in diameter wafer after the CCS growth routine of (*b*) Ru/Sr and (*c*) Ti/Sr. Note that Sr sets to 1 in Sr(Ti,Ru)$O_{3-\delta}$.

In this study, nine regions between two targets were scanned and analyzed to see the direct variation of Ru-doping level *in-situ*. The LAXS system we used for this study can scan the entire 37 regions over a 2-inch wafer. Figure S3 (a) shows the scan diagram of a 37-scanning point on a 2-in wafer. Figure S3 (b-c) displays the composition ratio of Ru/Sr and Ti/Sr adjusted by the sensitivity factor found from the RBS study. Note that these two 37-scanning data were taken on the CCS sample; after that, the growth with the LAXS routine of 9 scan points was completed.

Table S1. Composition percentage of the Ti-Ru phase spread by two targets, $SrTiO_3$ and $SrTi_{0.8}Ru_{0.2}O_3$, on a 2-in. wafer.

Scan position #	1	2	3	4	5	6	7	8	9
Ti (%)	70.4	70.4	73.6	83.2	80.0	85.6	84.8	90.4	98.4
Ru (%)	20.0	18.4	16.8	13.6	10.4	8.0	6.4	4.8	4.0

Composition percentage can be obtained from each composition ratio from LAXS method. Table S1 shows the composition percentage of the Ti and Ru phase spread at *B*-site in a perovskite structure between two targets, $SrTiO_3$ and $SrTi_{0.8}Ru_{0.2}O_3$, on a 2-in. wafer. Note that these values are achieved from Figure 5.
